# An immunocompetent patient presenting with severe nasal herpes simplex: a case report

**DOI:** 10.1186/1757-1626-2-9079

**Published:** 2009-11-23

**Authors:** Harry RF Powell, John Almeyda

**Affiliations:** 1 Ear Nose and Throat Department, West Middlesex University Hospital NHS Trust, Twickenham Road, Isleworth, Middlesex, TW7 6AF, UK; 2 The Royal Brompton Hospital, Sydney Street, London, SW3 6NP, UK

## Abstract

**Background:**

Cutaneous manifestations of common viral pathogens or disease processes more common in immunocompromised individuals need to be considered when assessing patients with unusual clinical presentations. To our knowledge this is the first published case of severe nasal herpes simplex infection in an immunocompetent individual.

**Case Presentation:**

A 27-year-old Burmese woman presented to the Accident and Emergency department with increasing facial pain and fever having sustained local trauma to her nose 7 days prior. Despite 5 days of treatment with oral Amoxicillin 500 mg TDS and topical Neomycin sulphate cream the patient developed blistering followed by de-epithelialisation of the nasal skin.

**Conclusion:**

Herpes simplex virus is very common, occasionally patients present with severe and potentially disfiguring lesions. This case is important for any sub specialists dealing with facial lesions. Early accurate diagnosis can improve outcome and reduce long-term morbidity.

## Introduction

Herpes simplex virus (HSV) typically presents as a localised vesicular eruption or ulceration a few days after exposure. There are a number of differential diagnoses including mycobacterium tuberculosis, wegener's granulomatosis, invasive fungal disease and leprosy. In immunocompromised patients the clinical presentation is often atypical and lesions may be chronic. Early diagnosis and appropriate management improve outcome. Depending on the anatomical location of the lesions referral to other specialties for example ENT, Dermatology, Maxillofacial and Ophthalmology may be indicated.

## Case presentation

A 27-year-old Burmese woman who had been living in the United Kingdom for 2 years presented to the Accident and Emergency department with increasing nasal pain and fever. 7 days prior to presentation she sustained local trauma to the side of her nose from a button. Over the next 2 days this became a blister covering most of her nose, which then burst. During the following three days the skin over her nose in the distribution of the nasociliary nerve broke down. From the second day she was being treated with oral Amoxicillin 500 mg TDS and topical Neomycin sulphate cream (prescribed by her General Practitioner). She was taking simple analgesia - Paracetamol and Ibuprofen as required.

**Figure 1:**
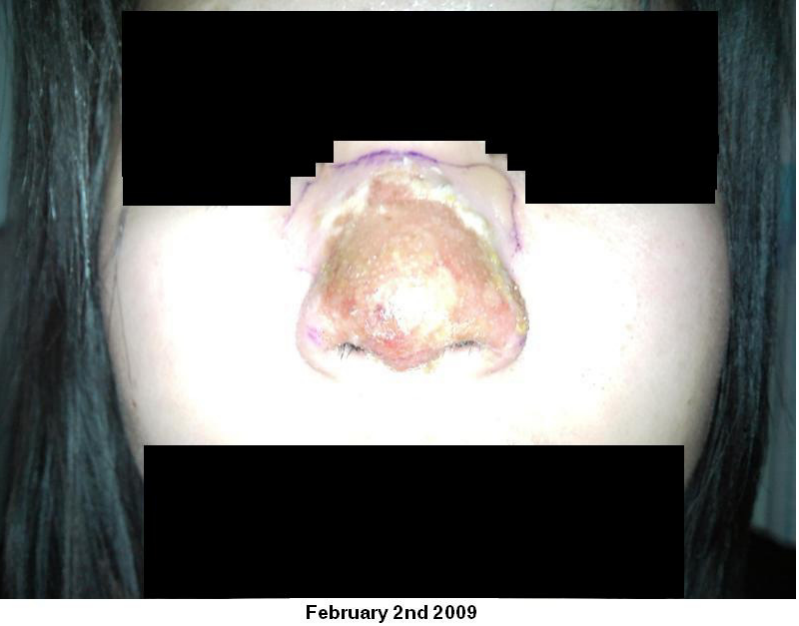
**9th February 2009**.

**Figure 2:**
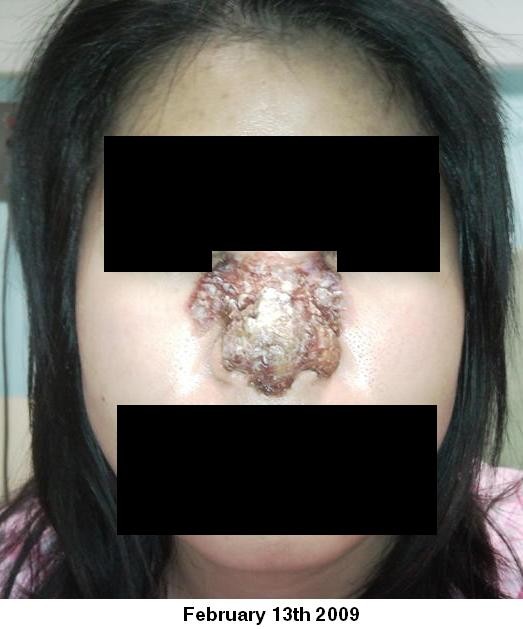
**13th February 2009**.

**Figure 3:**
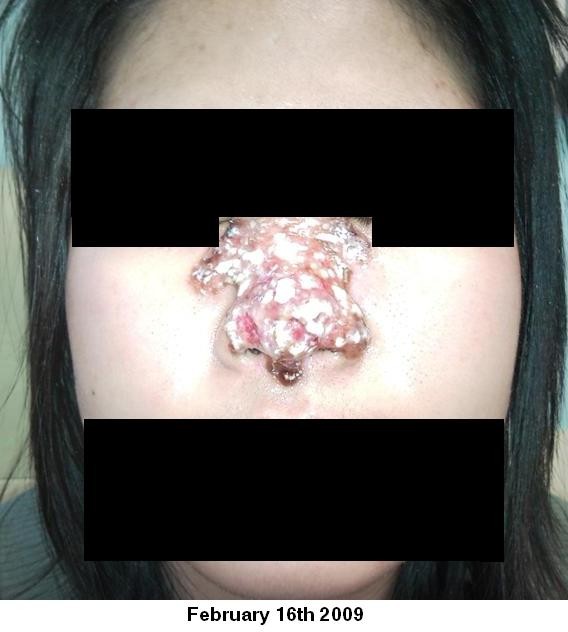
**16th February 2009**.

On examination there was erythema of the nose extending from the radix and both medial canthus to the alar rims. Within this there was a crusted, de-epithelialised paraesthetic area of skin approximately 3 × 2 cm square. There was no intranasal involvement on nasal endoscopy. She had a temperature of 37.9°C, white blood cell count of 10 (×1000/uL) and a C-reactive protein of 14 mg/L. Broad spectrum intravenous antibiotics were commenced. Figure 1, Figure 2, Figure 3 show clinical images of the progression of the infection and the significant skin damage that occurred.

On Day 1 a dermatology opinion was sought and a provisional diagnosis of herpes simplex made based on the clinical findings. The patient was started on 5 mg/kg intravenous Aciclovir TDS and isolated to a side-room as a precaution. Viral serology and a swab were sent for analysis and culture. On day 2, since there was no clinical improvement topical Aciclovir was added to the treatment regimen. Serologically herpes simplex virus (HSV) IGM and IGG antibodies were detected. The enzyme immunoassay (EIA) value for HSV IGG type 1 was 1.23 where a value <0.9 is negative, 0.9 - 1.1 is equivocal and >1.1 is positive. Diagnosis was confirmed by HSV 1 DNA detection on polymerase chain reaction (PCR) of the skin swab (2 weeks later).

**Figure 4:**
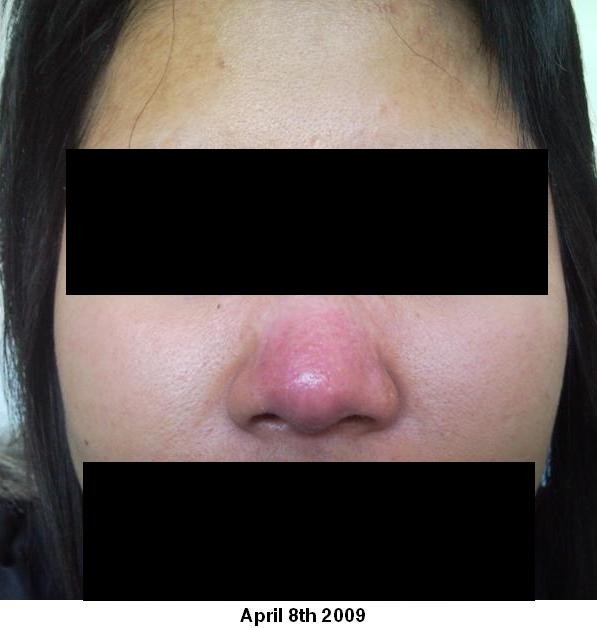
**8^th ^April 2009**. Taken in the outpatient department 2 months after hospital admission showing the patient's healed nasal skin.

At 8 week follow up there was complete re-epithelialisation and minimal scarring (see figure 4). The patient was instructed to avoid direct sunlight exposure and massage the area with Vaseline or E45 cream to help further improve the cosmetic appearance.

## Discussion

Herpes simplex virus has two subtypes; HSV1 is more common and generally causes ulceration around the mouth or nose known as cold sores whereas HSV2 is more likely to cause genital lesions. Via a similar pathological mechanism to varicella zoster, there is the possibility of latent and recurrent disease. Following primary infection the virus lies dormant in dorsal root ganglia of sensory nerve fibres. In response to a number of possible triggers the immune system is no longer able to contain latent viral replication and the virus is reactivated. Possible triggers are sunlight, immunosuppression, stress, fever and menstruation.

Diagnosis of HSV can be made on clinical presentation, direct fluorescent antibody assay, tissue culture, histopathology with immunohistochemistry, electron microscopy or PCR. Serology alone may give a high number of false positives as there is a high prevalence of HSV antibody in the normal population. Unusual presentations of HSV usually occur in immunocompromised individuals and in the literature have particularly been described in association with fludarabine chemotherapy and patients with lymphoma [1]. Rarely necrosis can occur and surgical debridement is necessary [2].

## Conclusion

To our knowledge this is the first published case of severe nasal herpes simplex infection in an immunocompetent individual. It is important that we are aware of this unusual nasal presentation of a common viral pathogen and are therefore able to instigate early anti-viral therapy to reduce associated morbidity.

## Consent

Written informed consent was obtained from the patient for publication of this case report and accompanying images. A copy of the written consent is available for review by the Editor-in-Chief of this journal.

## Competing interests

The authors declare that they have no competing interests.

## Authors' contributions

HP and JA were the registrar and consultant looking after the patient during their hospital admission. HP took the clinical photographs, gained the patient's consent, and wrote up the manuscript with constant guidance and support from JA. Both authors have read and approved the final manuscript.
